# Only Low Frequency Event-Related EEG Activity Is Compromised in Multiple Sclerosis: Insights from an Independent Component Clustering Analysis

**DOI:** 10.1371/journal.pone.0045536

**Published:** 2012-09-21

**Authors:** Hanni Kiiski, Richard B. Reilly, Róisín Lonergan, Siobhán Kelly, Marie Claire O'Brien, Katie Kinsella, Jessica Bramham, Teresa Burke, Seán Ó Donnchadha, Hugh Nolan, Michael Hutchinson, Niall Tubridy, Robert Whelan

**Affiliations:** 1 Trinity Centre for Bioengineering, Trinity College Dublin, Ireland; 2 Department of Neurology, St. Vincent's University Hospital, Elm Park, Dublin, Ireland; 3 School of Psychology, University College Dublin, Belfield, Dublin, Ireland; 4 Department of Psychiatry, University of Vermont, Burlington, Vermont, United States of America; Charité University Medicine Berlin, Germany

## Abstract

Cognitive impairment (CI), often examined with neuropsychological tests such as the Paced Auditory Serial Addition Test (PASAT), affects approximately 65% of multiple sclerosis (MS) patients. The P3b event-related potential (ERP), evoked when an infrequent target stimulus is presented, indexes cognitive function and is typically compared across subjects' scalp electroencephalography (EEG) data. However, the clustering of independent components (ICs) is superior to scalp-based EEG methods because it can accommodate the spatiotemporal overlap inherent in scalp EEG data. Event-related spectral perturbations (ERSPs; event-related mean power spectral changes) and inter-trial coherence (ITCs; event-related consistency of spectral phase) reveal a more comprehensive overview of EEG activity. Ninety-five subjects (56 MS patients, 39 controls) completed visual and auditory two-stimulus P3b event-related potential tasks and the PASAT. MS patients were also divided into CI and non-CI groups (n = 18 in each) based on PASAT scores. Data were recorded from 128-scalp EEG channels and 4 IC clusters in the visual, and 5 IC clusters in the auditory, modality were identified. In general, MS patients had significantly reduced ERSP theta power versus controls, and a similar pattern was observed for CI vs. non-CI MS patients. The ITC measures were also significantly different in the theta band for some clusters. The finding that MS patients had reduced P3b task-related theta power in both modalities is a reflection of compromised connectivity, likely due to demyelination, that may have disrupted early processes essential to P3b generation, such as orientating and signal detection. However, for posterior sources, MS patients had a greater decrease in alpha power, normally associated with enhanced cognitive function, which may reflect a compensatory mechanism in response to the compromised early cognitive processing.

## Introduction

Cognitive impairment (CI) affects nearly 65% of multiple sclerosis (MS) patients and can occur in the absence of physical disability [1]. Dysfunctions in speed of information processing, attention, memory and executive functions are most typically observed in MS patients [2], which have an adverse impact on daily life [3], [4]. It is important to recognise CI as early as possible and to monitor its course frequently [5]. Disease duration and disability level are usually poorly correlated with CI in MS [5], and the MRI lesions do not thoroughly explain differences in cognition of individual MS patients [6]. Neuropsychological tests in the MS Functional Composite [7], such as the Paced Auditory Serial Addition Test (PASAT; a difficult test of attention and working memory [8], [9]), are generally used to measure CI in MS. Nevertheless, the PASAT is influenced by practice effects [10], education, anxiety and physical ability which is usually impaired in MS [11], and therefore objective and reliable cognitive electrophysiological measures, which are not affected by these factors, have potential to complement the assessment of CI in MS.

### Scalp-based P3b ERP studies with MS patients

Prior electroencephalography (EEG) studies have concentrated on measuring scalp P3b event-related potential (ERP) and EEG time-frequency domain in MS patients [12], [13], [14], [15], [16], [17], [18], [19], [20], [21], [22]. The most common method to investigate the relationship of CI and ERPs is to employ a two-stimulus *oddball* task, in which occasional target stimuli have to be detected in a train of frequent irrelevant non-target stimuli. A *P3b* component is typically elicited approximately 300 ms after each target stimulus, with maximal amplitude over the parietal scalp area [23]. The neural generators in healthy controls of P3b have not been comprehensively delineated, not least because they are widespread in nature, but they include the parietal, frontal, and medial temporal lobes, the cingulate cortex, and the temporo-parietal junction [23], [24]. The P3b is considered to reflect context updating [23] and/or the categorization of task relevant events [25].

The majority of P3b ERPs studies in MS have reported prolonged P3b latencies and/or reduced P3b amplitudes in visual and/or auditory modality [12], [13],[15],[16],[17],[18],[19], although some studies [14], [19] have failed to find differences between MS patients and controls. The variation in the scalp-based P3b ERP results was most likely due to the relatively small sample sizes and the use of low-density EEG arrays. In our previous scalp-based high-density EEG study [22], with a relatively large number of subjects (N = 54), we showed that MS patients had reduced P3b amplitudes over frontal and centro-parietal scalp areas in the visual task, but there were no statistically significant differences in auditory modality. Furthermore, in a subsequent longitudinal high-density EEG analysis [26] we demonstrated visual modality to be more sensitive in detecting a greater reduction of P3b amplitudes over the centro-parietal scalp region in MS patients relative to controls over 12-month period. Moreover, both visual and auditory P3b amplitudes had decreased over the centro-parietal scalp area in MS patients after 12 Months. In brief, the visual modality appeared to be superior in detecting change in scalp-based analysis of P3b ERPs in MS patients.

Physiologically, EEG spectral power reflects the number of neurons that discharge synchronously (i.e. increased power implies increased synchronous firing of a group of neurons) [27]. *Theta* (4–8 Hz), *alpha* (8–13 Hz), and *beta* (14–30 Hz) EEG frequencies are associated with cognitive processing and behaviour [23], [28]. In particular, superior working memory performance is related to an increase in theta power and to a decrease in alpha power after an increase in task demands, such as the presentation of less frequent target stimuli in an oddball paradigm [27]. Theta oscillations are also related to information processing tasks, especially those of sustained attention and executive functions [27], [29], [30], [31], which are all essential for a successful oddball detection. Previous scalp-based and low-density spectral EEG studies on MS patients related to P3b ERP task performance have identified increased power of only beta and gamma bands in MS patients compared to controls over the whole scalp area, which was most prominent over frontal electrodes [21], and over anterior right hemisphere and bilateral posterior scalp regions [20].

### Beyond scalp ERP

Previous EEG studies of cognitive functioning in MS have only examined the differences between the mean ERPs of MS patients and controls [12], [13], [14], [15], [16], [17], [18], [19], [22], [26], [32]. The informativeness of ERPs is restricted due to the fact that they are generated by multiple, functionally distinct neural sources, which cause EEG potentials recorded on the scalp to overlap both temporally and spatially [33], [34]. Independent component analysis (ICA) is one of the most advanced methods to deal with the problem of spatiotemporal overlap of EEG. Its aim is to identify temporally independent and spatially fixed independent components (ICs) from high-density scalp EEG activities in which they are intermingled, yielding functionally distinct ICs which can be mapped onto 3D head space [35], [36], [37]. Through this inverse source modelling [37], the ICs reflect the origin of the EEG activations, whereas in the traditional scalp-based ERP analysis the recorded signals from each scalp electrode are assumed to be comparable with the signals from equivalently placed electrodes for all the subjects, thus not taking into account the physical differences of the brains of different people [37]. In contrast to other EEG source analysis methods (e.g., BESA) [38], ICA does not require *a priori* assumptions on the temporal dynamics or the spatial structure of the underlying processes. Moreover, ICA is a useful method for isolating artifactual EEG sources (such as eye blinks and muscle artifact), which are then removed, yielding a more accurate representation of neural activity [35].

Average ERPs between groups only capture the portion of the channel data that is phase-consistent at latencies relative to the time-locking events, which may result into a potential loss of information of the EEG activities [35], [37]. The aggregation of ICs into functionally equivalent groups provides an estimate of source EEG activities across subjects and conditions, thus reflecting more comprehensively the EEG activities [37]. Event-related spectral perturbations (ERSPs) and inter-trial coherence (ITCs) are superior methods for visualizing event-related EEG dynamics because they reveal a more comprehensive overview of the underlying EEG time-frequency properties than ERPs. ERSPs are the mean latency-by-frequency images that show the frequencies and latencies when mean changes in log power (dB) occur from a specific mean power baseline, showing them time-locked to experimental events [39]. ITCs measure the trial-to-trial phase consistency at each frequency and latency relative to a set of time locking events. If ITC is close to 0 at a given frequency and latency (relative to time-locking events) then the measured spectral phase is evenly distributed across trials, whereas if ITC is near 1, then the activity is reliably phase locked to the time-locking events [40], [41].

An IC clustering study of an auditory P3b task in healthy, young volunteers showed that IC clusters reflecting P3b activations had a decrease in alpha power post-target stimulus presentation [42]. To our knowledge, IC clustering of EEG data has not yet been utilised with a large sample (over 40 subjects), nor with a clinical group. IC clustering has potential to give a more thorough insight into how the changes in MS patients brains, due to demyelinating lesions and cortical atrophy, affect the speed of processing of the stimulus information.

## Methods

### Ethical approval

Ethical approval was obtained from the St. Vincent's University Hospital Ethics Committee. Written informed consent was obtained from all subjects.

### Hypotheses

Based on earlier findings on scalp-space P3b ERP analyses in MS [16], [22], [32], [43] and on spectral EEG studies relating frequency bands to cognitive functions utilised during P3b task performance, such as sustained attention and working memory [27], [29], [30], [31], it was hypothesized that MS patients versus controls and cognitively impaired MS (CI-MS) patients versus non- cognitively impaired MS (non-CI-MS) patients would show 1) reduced theta and a smaller decrease in alpha band after target stimuli, and 2) less consistency in IC inter-trial coherences (ITCs), over the expected P3b peak time period (200–400 ms). These differences were expected to be more prominent in the IC clusters located in the parietal brain areas which were associated with P3b activities in prior IC clustering studies [37], [42], [44], [45], and in visual modality based on the previous studies of our research group [22], [26].

### Subjects

Fifty-six MS patients (mean age = 44 years; range = 20–61 years) satisfying the revised McDonald criteria for MS [46] were recruited. The MS patients were age-matched with 39 healthy volunteers (mean age = 42 years; range = 28–67 years). Exclusion criteria included current use of benzodiazepines or neuroleptics (minimum suspension period of 7 days), a history of alcohol or drug misuse, head injury, or stroke. Table 1 displays the demographic and behavioural data of the subjects. Six patients had had a relapse within the previous year. Three MS patients were excluded from the further analysis of the PASAT performance groups due to missing information. The effect of years of education, age and gender on PASAT scores were controlled by employing a regression-based approach (described in detail in [47]) to obtain corrected PASAT Z-scores due to the differences and correlations between MS patients and controls in the years of education, age and gender. The MS patients were then chosen for an extreme group analysis based on their PASAT Z-score. In this analysis, the top third of the MS patients (N = 18) with highest PASAT Z-scores (mean = 0.91) denoted here as the non-cognitively impaired MS group (non-CI MS) were compared to the bottom third of MS patients (N = 18) with the lowest PASAT Z-scores (mean = −1.54) denoted here as the cognitively impaired MS group (CI MS).

**Table 1:** Demographical and behavioural data of subjects.

	MS (N = 56)		Control (N = 40)		Non-CI (N = 18)		CI (N = 18)	
Male/female	30/26		22/18		12/6		9/9	
	Mean	SD	Mean	SD	Mean	SD	Mean	SD
Age	44.14	9.87	42.47	9.89	45.17	7.72	40.12	9.55
Mean years of disease	11.64	8.59	N/A	N/A	11.50	8.52	10.60	6.92
Mean years of symptoms	15.81	10.26	N/A	N/A	14.22	8.30	15.72	11.63
Mean years of education	14.65	3.59	17.24	2.98	12.22	2.58	17.00	3.40
PASAT (Z-score)	−0.33	1.14	0.033	1.49	0.91	0.70	−1.54	0.54
Auditory P3b RT (<800 ms)	372.81	75.12	359.82	74.64	372.52	68.36	378.01	101.43
Visual P3b RT (<800 ms)	381.92	72.90	363.45	47.27	390.53	77.53	379.49	77.18

	Median	IQR	Median	IQR	Median	IQR	Median	IQR
EDSS	3.50	4.00	N/A	N/A	3.00	4.63	3.25	4.125
Hamilton	3.00	6.50	0.50	4.50	4.50	7.00	4.00	12.00

Note. Non-CI = MS patients with the highest PASAT Z-score, CI = MS patients with the lowest Z-score, PASAT = Paced Auditory Serial Addition Test, EDSS = Expanded Disability Status Scale, RT = reaction time, SD = standard deviation, IQR = interquartile range.

### Procedure

ERP data were recorded using the ActiveTwo Biosemi™ system, from 134 electrodes (128 scalp electrodes), organized according to the 10–5 system [48]. EEG data were digitized at 512 Hz. The vertical and horizontal electro-oculograms were recorded bilaterally from approximately 3 cm below the eye and from the outer canthi respectively. Participants were seated in a soundproofed, darkened room. The visual P3b paradigm consisted of blue circles, separated by an inter-stimulus interval of 2 s, presented for 205 trials in a pseudorandom order. Frequent non-target (probability = 0.8) and infrequent target (probability = 0.2) circles were 2 cm or 4 cm in diameter, respectively. The auditory P3b paradigm consisted of tones, separated by an inter-stimulus interval of 2 s, presented binaurally for 205 trials in a pseudorandom order. Frequent non-target (80%) and infrequent target (20%) tones were presented at 500 Hz and 1000 Hz respectively. Subjects were instructed to press a button as quickly as possible following a target stimulus. Order of modality and task were counterbalanced across subjects.

Subjects completed a neuropsychological measure of speed of cognitive processing, the standard 3-s Paced Auditory Serial Addition Test PASAT [8], [9]. The subjects sat with the examiner in a quiet room, and were asked to add consecutive single-digit numbers as they were presented on a compact disk and to respond orally with the accurate sum. Subjects were asked to perform calculations silently, without writing or using fingers, and a practice sequence was administered prior to the test. The standard PASAT form, consisting of 61 single digits with a 3 s inter-stimulus interval, was used. Raw PASAT score was based on the total number of correct responses from a maximum of 60 correct answers. Due to known correlations between PASAT raw scores and and demographic variables such as age [9], [49], [50], education and gender, the PASAT raw scores were corrected for demographic variables using Parmenter's [47] regression-based demographic corrections for MS patients, and the demographic corrected PASAT Z-scores were then utilised in the data analysis.

The subjects also completed the 21-item Hamilton rating scale for depression (HRSD-21) [51], and MS patients completed a measure of physical disability in MS, Kurtzke Expanded Disability Status Scale (EDSS) [52].

### Data analysis

Statistical analyses on demographical and behavioural data were completed using PASW Statistics 18.0 (Predictive Analytics SoftWare; SPSS Inc., Chicago, IL, USA, www.spss.com). Preliminary data processing employed the EEGLAB toolbox ([53]; http://sccn.ucsd.edu/eeglab) in conjunction with the FASTER plug-in (Fully Automated Statistical Thresholding for EEG artifact Rejection; [54]; http://sourceforge.net/projects/faster). The EEG data were bandpass filtered between 1 and 95 Hz, notch frequencies set to 50 and 60 Hz (the monitor refresh rate), average referenced across all scalp electrodes (appropriate when using a high-density EEG array), epoched from 500 ms pre-stimulus to 1000 ms post-stimulus and baseline corrected from 200 ms pre-baseline). The FASTER toolbox removed epochs with large artefacts (e.g., muscle twitch) and interpolated channels with poor signal quality. All pre-processing parameters for FASTER for this study are contained in Supplemental Material.

Independent component analysis (ICA) was used to obtain independent components (ICs) from scalp EEG activity in which they were mixed [55], [56]. This was performed using the *Infomax* algorithm [55]. The ICA has the effect of spatially filtering the data by using the information from in the EEG itself to separate scalp data from each active cortical source. Specifically, EEG channel recordings were arranged in a matrix of channels by time points, which was then submitted into ICA. ICA then found an ‘unmixing’ matrix *W* that linearly unmixed (i.e. decomposed) the multichannel EEG data (*x*) into a sum of maximally temporally independent and spatially fixed components *u* (i.e., *u = Wx* [37]). The rows of the resulting matrix *u* were the IC activations and its columns the time points of the input data [40]. Columns of the inverse matrix, *W^−1^*, gave the relative projection weights from each IC to each scalp electrode [37]. The auditory and visual P3 data were subjected to two separate ICAs because the tasks involved different sensory processes activating different brain areas at different latencies. Based on our prior experience with ICA (e.g. developing FASTER, a fully automated method for processing high-density EEG data; [54]) we found that applying separate ICAs was optimal.

FASTER [54] automatically identified artifactual (i.e., non-neural) ICs and removed them from the EEG data (note: the standard *z* = 3 threshold for IC rejection was lowered to *z* = 2 for the EOG channels). The remaining ICs were used in the further analyses. Next, inverse modelling of the individual IC scalp maps was performed to localize EEG sources related to P3b task performance [37]. An equivalent current dipole model was computed for each brain activity component map by using a four-shell spherical head model in the DIPFIT2 plug-in (http://sccn.ucsd.edu/eeglab/plugins.html), co-registered to the Biosemi coordinates via a Talairach transformation matrix. DIPFIT2 used automatic single dipole source localization algorithm for individual component maps in order to test the goodness-of-fit for modelling each IC scalp map with a single equivalent current dipole to quantify component quality. ICs were excluded from further analysis if 1) the best-fitting equivalent current dipole model had more than 15% residual variance from the spherical forward-model scalp projection (over all 128 scalp electrodes) and/or 2) the component's equivalent dipole was located outside of the model brain volume.

In order to acquire comprehensive source EEG activations related to P3b task, IC clusters were defined by means of a joint distance measure based on three dimensional (3D) dipole source locations and orientations, scalp maps, mean spectra (using a Fast Fourier Transform), and ERP, ERSP and ITC activations. The sliding windows in the frequency analysis were based on the Morlet wavelet transform (maximum 185 ms). Computation was performed on 100 frequencies from 3–256 Hz.

Principal components analysis was utilised for pre-clustering with the relative weight = 1 for all of the measures, except for dipoles which was = 10. A K-means method was employed in IC clustering, and used Euclidean distances between the ICs to find first IC cluster centroids, and then include each IC observation into a cluster with the nearest mean. Outlier components of more than 2 standard deviations from any of the centroid properties of IC clusters were assigned to specific outlier IC clusters that were excluded from further analyses. On average, 26.8% of ICs were classified as outliers. The optimum number of final clusters was determined by starting with a small number of clusters (the smallest number in which activation properties were not merged across clusters) and then increasing the number of clusters by one. Following the addition of an extra cluster, if IC dipole groups were divided into two but the other IC properties (and the differences between the groups and conditions) remained approximately the same then the maximum number of clusters was deemed to have been exceeded. In this way, 4 distinct IC clusters were identified in the visual condition and 5 IC clusters in the auditory condition.

Permutation statistics were applied to each cluster [57], [58], in conjunction with the false discovery rate correction for multiple comparisons [59]. Permutation statistics were chosen as they are non-parametric and therefore do not rely on an a priori model of the data distribution [57], [58]. In permutation-based approaches sets of simulated sample distributions are generated by randomly shuffling the data across trials and/or subjects. Consequently, the shuffled distributions will have all features of the original data except the effect to be tested [37], [57], [58]. The false discovery rate is the expected proportion of erroneous rejections among all rejections and it can be used to control the familywise error rate (i.e. the probability of having at least one type I error among the entire set of tests conducted) [59]. In order to correct for the number of clusters per condition, a Bonferroni cut-off was also applied, which involves dividing the *p*-value threshold by the number of comparisons performed [57]. Thus, the corrected significance cutoff was *p* = 0.0125 (i.e., .05/4) for the 4-cluster solution in the visual condition, and *p* = 0.01 (i.e., .05/5) for the 5-cluster solution in the auditory condition. The group statistic t-values which are below the significance cutoff are displayed in red in the ERSP and ITC subfigures showing the statistically significant differences between the groups relative to time and frequency.

In order to provide a comparison with the IC clustering method, an analysis of the scalp EEG data was also performed. The data pre-processed through FASTER were grand-averaged and statistical analysis of ERP, ERSP and ITC conducted at Pz. The false discovery rate correction for multiple comparisons [59] was utilised.

## Results

### Behavioral results

MS patients and controls did not differ in age or in reaction times (p>.05). MS patients had significantly higher scores in the HRSD-21 than controls (p<.001). PASAT score did not correlate with HRSD-21 score, but it correlated with EDSS score (p<.05). Controls had more years of education and higher PASAT scores than MS patients (p<0.001). As expected, the raw PASAT scores correlated with years of education in a sample of all subjects, with age and years of education in the MS patient sample, and with years of education and gender in the control sample.

PASAT raw scores were converted to demographically corrected scores (PASAT Z-scores: described in detail in [47]) and the MS patients were then grouped into two extreme groups based on their PASAT Z-score: the MS patients with the highest PASAT Z-scores or the non-cognitively impaired MS group (non-CI MS), and the MS patients with the lowest PASAT Z-scores or the cognitively impaired MS group (CI MS) as detailed in Methods.

The non-CI MS and CI MS groups did not differ in age, disease duration, reaction times, EDSS scores or HRSD-21 (p>.05). The PASAT Z-scores did not correlate with EDSS scores, with mean years of symptoms, or with mean years of disease but the CI MS group had more years of education than the non-CI MS group.

### MS patients vs. controls

**Figure 1:**
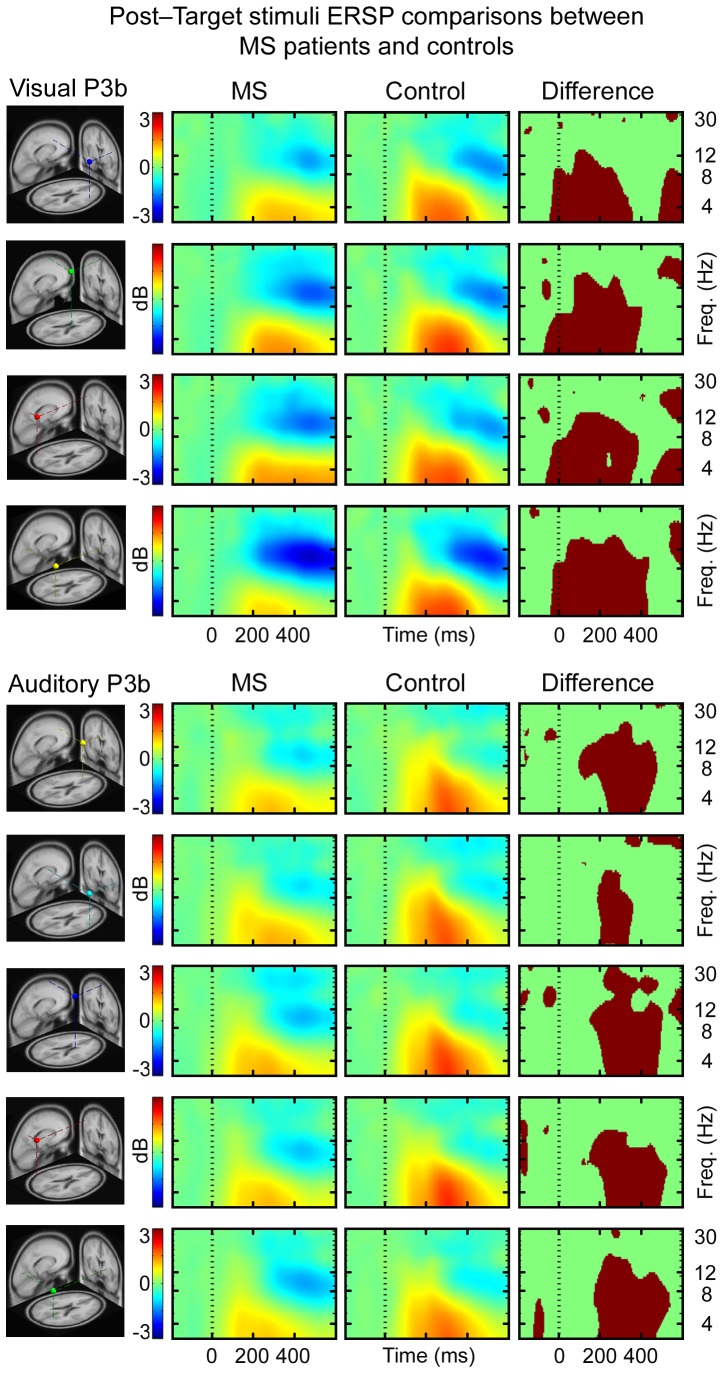
The mean ERSPs time-locked to the presentation of target stimuli in visual and auditory conditions are showing IC cluster mean differences in log spectral EEG power (dB) relative to log power in pre-stimulus EEG baseline. Red areas indicate an increase in power and blue areas a decrease in power. Statistical significance is illustrated by red/green frames beside ERSP activation frames, in which red areas signify statistically significant (p<0.0125 for visual condition and p<0.01, controlled for multiple comparisons) differences between the MS patients and controls in time and in log spectral power.

**Figure 2:**
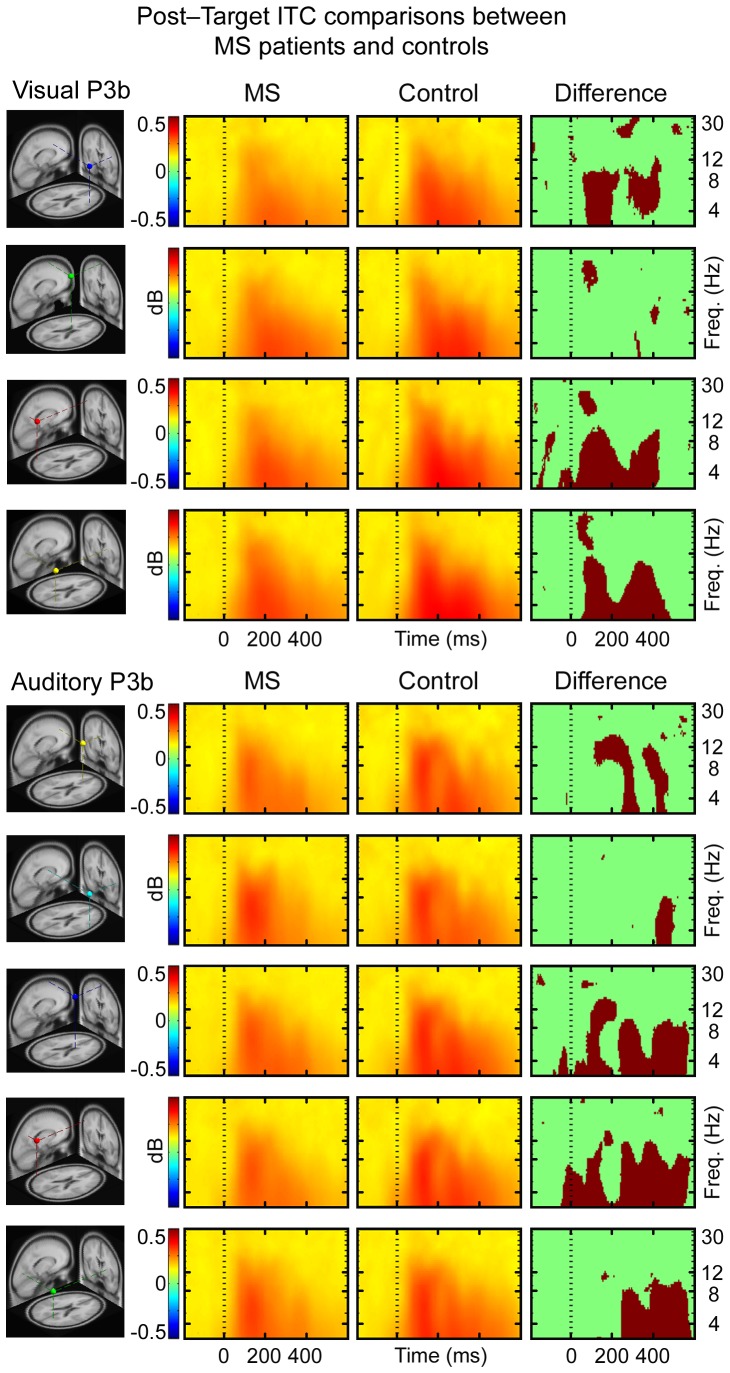
The ITC showing consistency between the trials and the degree of phase-locking to the target stimuli. Highest event-related phase consistencies for each condition are plotted in red, and lowest in green (range 0 to 1, no consistency to full consistency respectively). Statistical significance is illustrated by red/green frames, in which red areas signify statistically significant (p<0.0125 for visual condition and p<0.01 for auditory condition, controlled for multiple comparisons) differences between MS patients and controls in time and in log spectral power.

The ERSPs of all of the IC clusters revealed significantly less increase in theta power and a greater decrease in alpha power at 200–400 ms post-target stimulus for the MS patients when compared to the controls in all of the IC clusters for both visual and auditory conditions. In visual condition, there was less increase in theta power and a greater decrease in alpha power 0–200 ms for MS patients relative to controls (Figure 1). The inter-trial consistency was lower for MS patients compared to controls over theta frequencies in right frontal, right parietal and left parietal IC clusters at 0–400 ms after target stimuli presentation of visual condition; and in central and left parietal IC clusters at 0–600 ms and in frontal and right parietal IC cluster at 200–600 ms of auditory condition (Figure 2). The grand mean IC cluster scalp maps are displayed in Figure S6.

After non-target stimuli in the visual condition, the MS patients showed less increase in theta power 0–600 ms and a greater reduction in alpha power 200–600 ms compared to the controls in all ERSPs of IC clusters. In the auditory condition the MS patients showed less increase in theta, alpha and beta power around 0–200 ms and less decrease in theta, alpha and beta power 200–600 ms relative to controls in the left parietal IC cluster. The MS patients showed less increase in theta power at 100–200 ms in the frontal, right temporal and central IC clusters compared to controls (Figure S1). The inter-trial consistency was lower for MS patients relative to controls in the theta band from 0–600 ms in all of the IC clusters, and from 200–600 ms in the alpha band in left and right parietal IC clusters after visual non-target stimuli presentation; and over theta and alpha bands at 0–400 ms in frontal, central, left parietal and right parietal IC clusters, and at 200–400 ms in right temporal IC cluster of the auditory condition (Figure S2). The grand mean IC cluster scalp maps are displayed in Figure S7.

### Non-CI MS vs. CI MS

**Figure 3:**
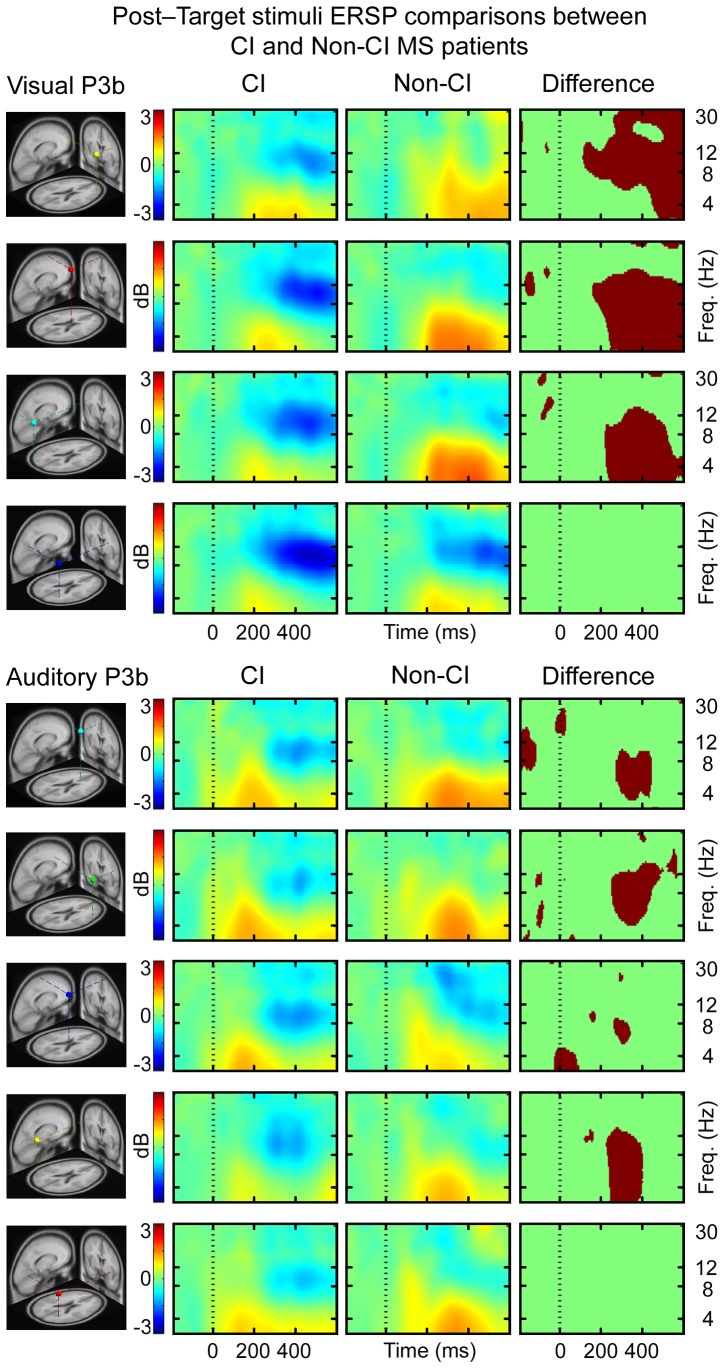
The mean ERSPs time-locked to the presentation of target stimuli in visual and auditory conditions are showing IC cluster mean differences in log spectral EEG power (dB) relative to log power in pre-stimulus EEG baseline. Red areas indicate an increase in power and blue areas a decrease in power. Statistical significance is illustrated by red/green frames beside ERSP activation frames, in which red areas signify statistically significant (p<0.0125 for visual condition and p<0.01, controlled for multiple comparisons) differences between CI MS patients and the non-CI MS patients in time and in log spectral power.

**Figure 4:**
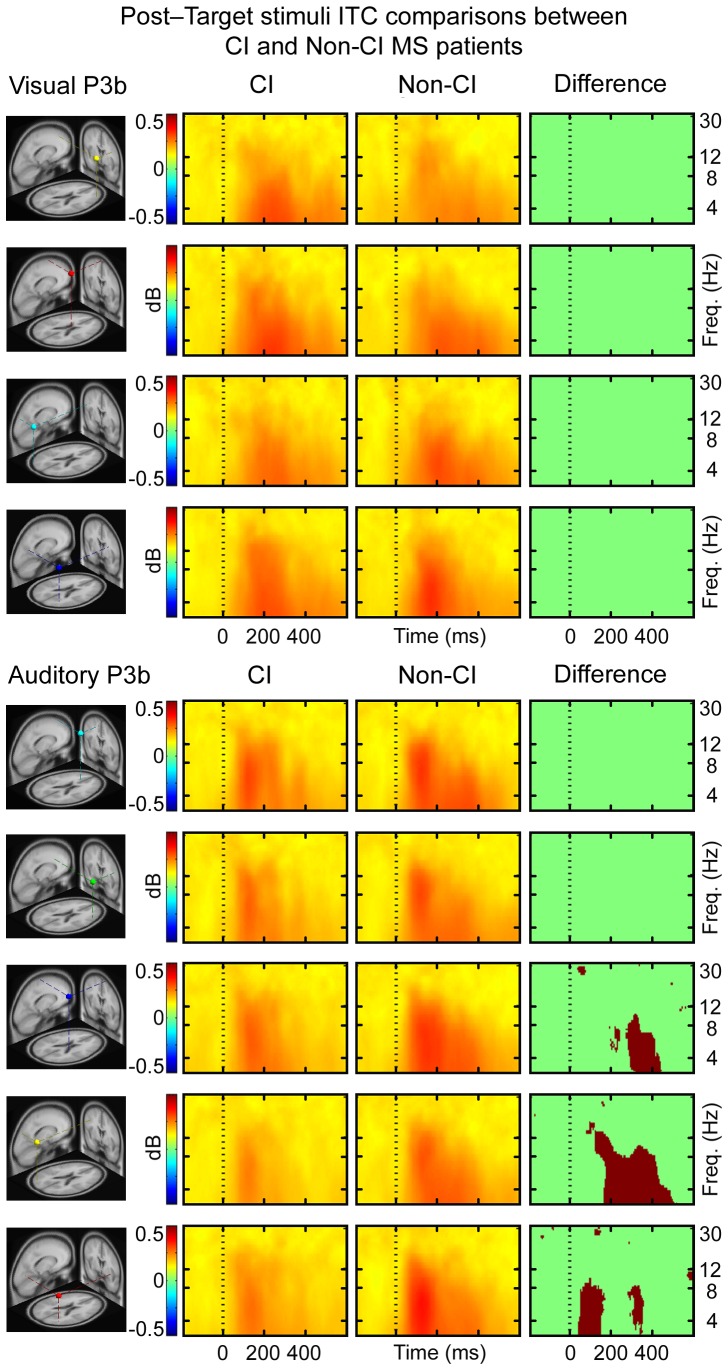
The ITC showing consistency between the trials and the degree of phase-locking to the target stimuli. Highest event-related phase consistencies for each condition are plotted in red, and lowest in green (range 0 to 1, no consistency to full consistency respectively). Statistical significance is illustrated by red/green frames, in which red areas signify statistically significant (p<0.0125 for visual condition and p<0.01 for auditory condition, controlled for multiple comparisons) differences between the CI MS patients and the non-CI MS patients in time and in log spectral power.

ERSP measures of the visual condition showed that CI MS patients had less theta, alpha and beta power at 200–600 ms post-target stimuli in the right frontal IC cluster relative to non-CI MS patients. In central and left parietal IC clusters CI MS patients showed reduced theta power at 200–600 ms post-target stimulus, relative to non-CI MS patients. The CI MS patients showed a greater decrease in alpha power in at 200–600 ms post-target stimulus compared to non-CI MS patients in right frontal, central and left parietal IC clusters (Figure 3, upper panel). The ERSP measures of the auditory condition indicate that CI MS patients had reduced theta power at 200–500 ms post-target stimuli compared to non-CI MS patients in left frontal, right frontal, and left parietal IC clusters (Figure 3, lower panel). CI MS patients had less inter-trial consistency compared to non-CI MS in the central and left parietal IC clusters in the theta band at 200–400 ms post-target stimuli (Figure 4, lower panel). There were no differences between the groups in ITC measures (Figure 4, upper panel) for the visual condition. The grand mean IC cluster scalp maps are displayed in Figure S8.

There were no clear differences in ERSP between the groups after the presentation of visual non-target stimuli, but in auditory condition there were subtle differences in theta, alpha and beta power at 0–600 ms in central and left parietal IC cluster (Figure S3). The only difference in ITC measures was in the left parietal IC cluster of auditory condition, in which CI MS patients had less inter-trial consistency compared to non-CI MS patients in theta band from 200–400 ms, and in the theta and alpha bands from 0–200 ms (Figure S4). The grand mean IC cluster scalp maps are displayed in Figure S9.

Talairach coordinates, Brodmann areas and associated brain regions of the IC cluster centroids of all of the comparisons are presented in Table 2. The numbers of independent components and subjects of each group included in clusters are reported in Table S1.

**Table 2:** Number of subjects and components in each IC cluster, and Talairach coordinates with associated Brodmann areas and brain regions of each IC cluster centroid.

	Number of subjects	Number of components	Talairach coordinates			BA	Associated brain regions
			x	y	z		
**MS vs. C**							
**Visual**							
Right frontal IC cluster	59	545	46	20	26	46	Frontal Lobe, Middle Frontal Gyrus
Central IC cluster	59	612	−1	3	67	6	Left Cerebrum, Frontal Lobe, Superior Frontal Gyrus
Right parietal IC cluster	92	747	21	−65	−14	31	Limbic Lobe, Posterior Cingulate
Left parietal IC cluster	54	475	−54	−59	31	40	Parietal Lobe, Supramarginal Gyrus
**Auditory**							
Frontal IC cluster	66	519	−4	36	26	32	Left Cerebrum, Limbic Lobe, Anterior Cingulate
Right temporal IC cluster	70	518	60	4	14	6	Frontal Lobe, Precentral Gyrus
Central IC cluster	65	683	11	6	67	6	Right Cerebrum, Frontal Lobe, Superior Frontal Gyrus
Right parietal IC cluster	93	706	20	−69	9	30	Occipital Lobe, Cuneus
Left parietal IC cluster	65	574	−59	−52	29	40	Parietal Lobe, Supramarginal Gyrus
**CI vs. Non-CI**							
**Visual**							
Right frontal IC cluster	21	170	45	39	30	9	Frontal Lobe, Superior Frontal Gyrus
Central IC cluster	22	227	−2	2	67	6	Left Cerebrum, Frontal Lobe, Superior Frontal Gyrus
Right parietal IC cluster	35	215	26	−59	21	31	Limbic Lobe, Posterior Cingulate
Left parietal IC cluster	20	139	−43	−79	25	19	Temporal Lobe, Middle Temporal Gyrus
**Auditory**							
Left frontal IC cluster	27	211	−8	36	47	8	Frontal Lobe, Superior Frontal Gyrus
Right frontal IC cluster	28	219	49	25	23	46	Frontal Lobe, Middle Frontal Gyrus
Central IC cluster	25	220	15	−19	68	6	Right Cerebrum, Frontal Lobe, Precentral Gyrus
Right parietal IC cluster	33	217	32	−66	0	19	Occipital Lobe, Lingual Gyrus
Left parietal IC cluster	28	236	−29	−80	39	19	Parietal Lobe, Precuneus

Note. BA = Brodmann area, IC = independent component, MS = MS patients, C = controls, CI = CI MS patients, non-CI = non-CI MS patients.

**Figure 5:**
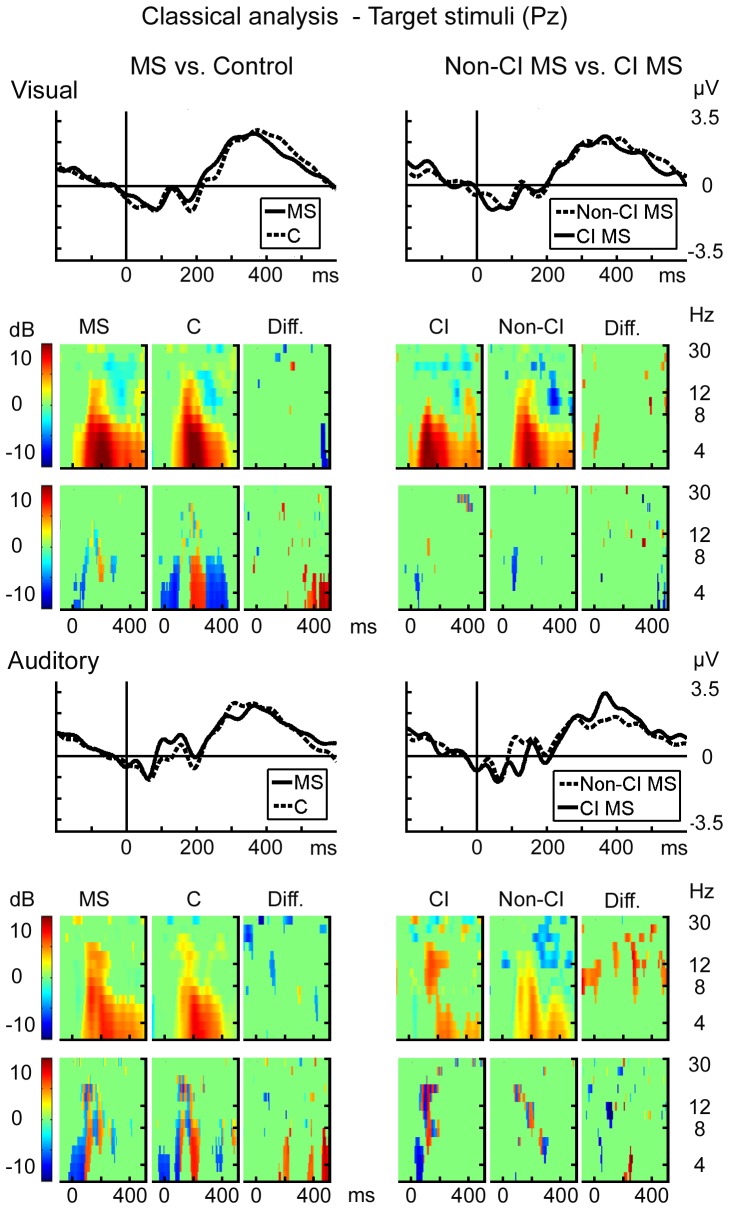
The scalp-based analysis of A) ERP, B) topography, C) ERSP, C) ITC of post-target activations at Pz in visual and auditory condition; comparison of MS patients and controls on the left side, and of CI and non-CI MS patients on the right side. Statistically significant (p<.05) differences in ERP indicated by red, and in the ERSP and ITC difference frames by non-green areas relative to time and log spectral power.

Scalp-EEG analysis results are presented in Figure 5 and in Figure S5. There were no differences when the target responses were compared (Figure 5). The MS patients had a larger P2 peak after non-target stimuli relative to controls in auditory P3b (Figure S5).

## Discussion

### The results of the present study

IC clustering of source EEG activities related to the P3b ERP is a novel approach for investigating CI in MS. To our knowledge, ours is the first study to employ IC clustering method with a clinical group and it is the largest EEG IC clustering study to date with 95 subjects (56 MS patients, 39 controls). In general, ERSP measures showed MS patients to have reduced theta power relative to controls. A similar pattern was observed for CI MS patients vs. non-CI MS patients. Furthermore, alpha band responses were not diminished for the MS patients (and the CI-MS patients), contrary to our hypotheses.

The presentation of the target stimulus during an oddball task represents an increase in task demand, which, in cognitively intact individuals typically resulting in an increase in theta power and a decrease in alpha power post-stimulus presentation [27]. Theta oscillations are related to a number of information processing tasks, such as those requiring sustained attention and with high executive demands [30], [31], [60]. Later theta oscillations have also been suggested to reflect cortico-hippocampal feedback loops related to the evaluation of stimuli that become active after physical or context deviations and are followed by controlled processing in frontal cortex [61]. The extent of theta synchronization has been related to good working memory performance [27], [28]. In MS patients, a reduction in theta power may reflect the disruption of long-distance neural synchronization (likely due to intervening lesions) which is thought to primarily occur in lower frequency bands [28], thus compromising early processes such as orienting and signal detection. This notion was reflected by the smaller increase of theta and alpha power in MS patients during early sensory processing (0–200 ms)

Interestingly, MS patients and CI MS patients had a greater decrease in alpha power, which was contrary to our hypotheses. This may indicate that these groups were able to recruit more attentional resources later in the processing stream in order to partially compensate for the comprised earlier cognitive processes. In contrast, controls, who completed the earlier processing adequately, did not have to allocate any such additional resources. This result is concordant with the tendency of MS patients to be impaired in *speed* of information processing, rather than an inability to process information *per se* [62]. In other words, given adequate time, MS patients can complete cognitively demanding tasks.

### Comparison with previous studies

The two-stimulus oddball paradigm is one of the most popular ERP paradigms, and scalp-based EEG studies have reported P3b to be reduced in amplitude and/or delayed in latency in MS and indicated P3b related activations to occur over large parietal (and also over frontal and central) scalp regions [12], [13], [15], [16], [17], [18], [19], [22]. Most of these studies [12], [13], [16], [17], [22] have also observed late P3b peaks in MS patients, which in part explains the broad time range (200–600 ms) observed in the present study. Another factor influencing the timing of the P3b is age [63], and this study included a range of ages (MS SD = 9.87 years; control SD = 9.89), and therefore it is plausible that the age differences contributed to the range of the P3b.

The results of the present study differ from a prior study in this area (albeit one that utilized low-density, scalp-based spectral EEG), which reported increased power of higher frequencies (beta and gamma bands) in MS patients compared to controls over the whole scalp area after target-stimuli presentation. This effect was most prominent over frontal electrodes [21], and around anterior right hemisphere and bilateral posterior scalp regions [20]. However, only MS patients in the early disease stage and therefore potentially with less CI were included in [20], [21]. In contrast, the present study included patients with a wider range of CI, and also investigated the differences between patient groups divided based on their cognitive performance. Furthermore, the present results are more consistent with previous spectral studies on cognition [27], [29], [30], [31], [62] that have reported theta power changes due to increased demands on cognitive processes. It is possible that theta power reduction is a feature of more advanced cognitive impairment.

The neural generators of (frontal-midline) theta oscillations have been reported to be located in the anterior cingulate in various EEG source analyses (see [64]), which is thought to be one of the sites for P3b ERP generation and in general to have important role in the networks contributing to cognitive function. Nevertheless, other studies indicate the generators of theta include also regions including occipital/parietal and temporal cortices (e.g. [65]). In the present study differences between MS patients and controls were observed in extensive areas.

In previous scalp-based P3b ERP studies of our research group we found the visual modality to be more sensitive in identifying differences between MS patients and controls [22], [26]. Interestingly, in the present study, IC clustering did not show the visual modality to be superior to auditory condition in detecting differences between MS patients and controls as differences in theta and alpha power in broad brain areas were visible across both modalities. On the other hand, IC clustering on the two PASAT performance groups revealed the largest modality difference to be in visual modality.

After the presentation of non-target stimuli, controls demonstrated a greater increase in theta power compared to MS patients, and MS patients a greater decrease in alpha power relative to controls in all of the IC clusters in both modalities. These differences were apparent in the time-frame of P2 ERP component. Relatively high theta power during resting-state EEG has been linked to effective cognitive function, especially to processes related to sustained attention [64], [66]. Therefore, the apparent baseline differences between controls and MS patients may reflect the underlying differences in the background EEG due to the difficulties in maintaining sustained attention which is required for a successful P3b task performance. Moreover, it can be proposed that the MS patients have less effective modulatory processes changing the responsiveness of large neuronal ensembles in a context-sensitive manner [67]. Furthermore, this possibly reflects the wide-spread underlying difficulties in information processing functions in the brains of MS patients which are likely to be affected by several lesions caused by the focal loss of myelin. On the other hand, there were no differences between the two PASAT performance MS groups in the visual condition, and the only difference between the groups was in left parietal IC cluster in the auditory condition. The lack of differences between the PASAT performance MS groups is most likely due to the fact that their background EEGs are relatively similar, and it can be suggested that their differences in cognitive function are not greatly related to sustained attention.

The ICA and IC clustering methods has several advantages over the scalp-based EEG analysis. Firstly, the IC clustering method aggregates ICs into functionally equivalent groups and estimates the source EEG activities across subjects and controls whereas the scalp-based ERPs between groups only capture the portion of the channel data that is phase-consistent at latencies relative to the time-locking events leading to a potential loss of information on the activities [35], [37]. Furthermore, IC clustering reflects more accurately the spatiotemporal features of the EEG activations, as the scalp-based EEG is a sum of temporally and spatially overlapping channel activities originating from multiple, functionally distinct neural sources and non-brain sources [33], [34]. In fact, the independent component analysis is regularly utilized to isolate and remove artifactual EEG sources (such as eye blinks, muscle artifacts) from the EEG data, which yields a more accurate representation of neural activity compared to scalp-based ERPs with artifacts [37], [54]. Finally, the ICA methods estimate the origin of the activation (inverse source modelling) whereas scalp-based ERP assumes the recorded signals of the scalp electrodes to be comparable with the signals from equivalently placed electrodes for all the subjects, thus not taking into account the physical differences of the brains of different people (e.g. the differences in the orientations of cortical gyri and sulci causing different projections of exactly equivalent cortical sources [37]. For these reasons, the ERP P300 and ICA approach to EEG analysis are qualitatively different, and must be interpreted differently. It is also noteworthy that the topographies of ICs (Figures S6, S7, S8, S9) are not as informative as 3-D locations of the equivalent dipoles as minor differences across subjects in the orientation of equivalent dipoles for a set of equivalent ICs can produce different scalp maps, and as the ICs are aggregated into clusters according to their *functional* similarity despite apparent scalp map differences Therefore, the typical centro-parietal positivity typically seen with a scalp-based ERP approach may not be seen following summation of ICs. Moreover, ICA and IC clustering methods do not require *a priori* assumptions [35], [37], which is an issue in other EEG source analysis methods such as BESA [38]. However, ICA approaches do rest on a number of assumptions, and future research (perhaps using simultaneous EEG and functional magnetic resonance imaging) should attempt to quantify the accuracy of IC spatial estimations.

On the whole, IC clustering enables a more comprehensive understanding of the dynamic EEG source activations underlying EEG recorded during cognitive performance which are likely to be missed during analysis of traditional scalp-based EEG data alone [35], [37]. This notion is bolstered by the results of the scalp-based ERP, ERSP and ITC analysis in the present study, which did not reveal large differences among groups. The differences observed between the results from the IC earlier). Moreover, the averaging of the scalp-based event-related activations most likely does not reflect all the ongoing EEG activations as it captures only the portion of the scalp channel data that is phase-consistent at latencies relative to the time-locking events [35], [37]. Therefore relevant information may be lost [35], and as a consequence there are no statistically significant differences in scalp-ERPs found between the groups. Furthermore, the IC clustering avoids (at least in part) the vagueness of spatiotemporal overlap inherent in scalp-based EEG recordings by aggregating the ICs with spatiotemporal features into functionally equivalent groups and thus estimating the *source* EEG activities. The group differences were not a result from a mere selection of specific ICs, as ERP activations recomposed of ICs in each IC cluster and back-projected to Pz did not show similar clear group effects to IC clustering results (see Figures S10, S11). In addition, the EEG signals recorded on the scalp originate from multiple, functionally distinct neural and non-neural sources leading to a considerably high level of noise (besides spatiotemporal overlap) [35], which may mask the underlying differences between the groups. The differences in the results may also stem from the fact that physical characteristics and projection orientations from the cortex to the scalp differ across individual brains, which violates one of the main assumptions inherent in scalp-based ERP analysis that presume the position of an electrode to reflect the neural activities directly underneath it and the task-related EEG activations in the same location to be similar across different subjects. These factors may therefore have led to the lack of statistically significant differences even though there would be a real underlying difference or a trend between the groups.

Physiologically, EEG signals originating from the brain are thought to be associated with near-synchronous field activities within a connected patch of cortical pyramidal cells sharing a common alignment near-perpendicular to the cortical surface [68]. The maximally temporally independent component activities with near-dipolar scalp projections may represent physiologically distinct brain sources that can be associated with field activity partially or fully synchronized across a cortical patch (or possibly across and between two anatomically well-connected patches).Thus, the individual ICs and IC clustering method hold promise to estimate more accurately EEG source locations, which in conjunction with cognitive electrophysiological experiments such as the oddball paradigm may give insight into the source and electrophysiological activities related to information processing speed and cognitive performance. The development of similar methodology is an important aim in studies with neurological conditions as it may offer better understanding of the brain location(s) where the changes impacting cognitive function are occurring. In the present study, the widespread reduction in theta and alpha power in MS patients was obvious post-target stimuli whereas the prior scalp-based P3b ERP studies have reported varied results of latency, amplitude and topography differences in MS patients relative to controls.

Objective, reliable EEG methods such as IC clustering may have potential to aid the detection and monitoring of cognitive impairment in MS, and therefore to complement generally utilised neuropsychological assessment. The relationship between pathological changes in the brain white and grey matter, neurophysiological and neuropsychological cognitive function is imprecisely defined in MS [69], and should be examined in future studies. Inclusion of a full neuropsychological battery may help to define better the relationships of specific cognitive domains with EEG and/or MRI findings, as may the use of more complex oddball paradigms [70]. Moreover, future studies could explore the informativeness of more advanced EEG data analysis methods such as distributed source modelling incorporating EEG activations and structural information from individual subjects' MR images. In addition, longitudinal studies of EEG scalp and source activities spanning over several years are required to determine if EEG and ERPs have utility in predicting the changes in cognitive function in MS.

## Supporting Information

Figure S1 **The mean ERSPs time-locked to the presentation of non-target stimuli in visual and auditory conditions are showing IC cluster mean differences in log spectral EEG power (dB) relative to log power in pre-stimulus EEG baseline.** Red areas indicate an increase in power and blue areas a decrease in power. Statistical significance group and condition main effects, and interaction effect, are illustrated in red/green frames beside ERSP activation frames, in which red areas signify statistically significant (p<0.0125 for visual condition and p<0.01, controlled for multiple comparisons) differences between the MS patients and controls in time and in log spectral power.
(TIF)

Figure S2 **The ITC showing consistency between the trials and the degree of phase-locking to the non-target stimuli.** Highest event-related phase consistencies for each condition and group are plotted in red, and lowest in green (range 0 to 1, no consistency to full consistency respectively). Statistical significance is illustrated by red/green frames, in which red areas signify statistically significant (p<0.0125 for visual condition and p<0.01 for auditory condition, controlled for multiple comparisons) differences between MS patients and controls in time and in log spectral power.
(TIF)

Figure S3 **The mean ERSPs time-locked to the presentation of non-target stimuli in visual and auditory conditions are showing IC cluster mean differences in log spectral EEG power (dB) relative to log power in pre-stimulus EEG baseline.** Red areas indicate an increase in power and blue areas a decrease in power. Statistical significance group and condition main effects, and interaction effect, are illustrated in red/green frames beside ERSP activation frames, in which red areas signify statistically significant (p<0.0125 for visual condition and p<0.01, controlled for multiple comparisons) differences between CI MS patients and the non-CI MS patients in time and in log spectral power.
(TIF)

Figure S4 **The ITC showing consistency between the trials and the degree of phase-locking to the non-target stimuli.** Highest event-related phase consistencies for each condition and group are plotted in red, and lowest in green (range 0 to 1, no consistency to full consistency respectively). Statistical significance is illustrated by red/green frames, in which red areas signify statistically significant (p<0.0125 for visual condition and p<0.01 for auditory condition, controlled for multiple comparisons) differences between the CI MS patients and the non-CI MS patients in time and in log spectral power.
(TIF)

Figure S5 **The scalp-based analysis of A) ERP, B) topography, C) ERSP, C) ITC of post–non-target activations at Pz in visual and auditory condition; comparison of MS patients and controls on the left side, and of CI and non-CI MS patients on the right side.** Statistically significant (p<.05) differences in ERP indicated by red, and in the ERSP and ITC difference frames by non-green areas relative to time and log spectral power. Plots of the topographical responses 2-D circular view (looking down at the top of the head). Channel locations below head center are shown in a ‘skirt’ outside the cartoon head. Nose is at top of plot; left is left; right is right.
(TIF)

Figure S6 **The grand mean topographies of MS patients and controls for each IC cluster post-target in visual and auditory conditions.**
(TIF)

Figure S7 **The grand mean topographies of MS patients and controls for each IC cluster post-non-target in visual and auditory conditions.**
(TIF)

Figure S8 **The grand mean topographies of CI and non-CI MS patients for each IC cluster post-target in visual and auditory conditions.**
(TIF)

Figure S9 **The grand mean topographies of CI and non-CI MS patients for each IC cluster post-non-target in visual and auditory conditions.**
(TIF)

Figure S10 **The EEG-scalp analysis of recomposed ICs included in the IC clustering analysis.** A) ERP, B) mean topography, C) ERSP, D) ITC of post-target activations at Pz in visual and auditory condition; comparison of MS patients and controls on the left side, and of CI and non-CI MS patients on the right side. Statistically significant (p<.05, FDR corrected) differences in ERP indicated by red, and in the ERSP and ITC difference frames by non-green areas relative to time and log spectral power.
(TIF)

Figure S11 **The EEG-scalp analysis of recomposed ICs included in the IC clustering analysis.** A) ERP, B) mean topography, C) ERSP, D) ITC of post-non-target activations at Pz in visual and auditory condition; comparison of MS patients and controls on the left side, and of CI and non-CI MS patients on the right side. Statistically significant (p<.05, FDR corrected) differences in ERP indicated by red, and in the ERSP and ITC difference frames by non-green areas relative to time and log spectral power.
(TIF)

Table S1 **The number of subjects and independent components from each groups in each IC cluster.**
(DOCX)
