# Supplementary figures and images for: Only Low Frequency Event-Related EEG Activity Is Compromised in Multiple Sclerosis: Insights from an Independent Component Clustering Analysis
Source: PLoS One. 2012 Sep 21;7(9):e45536. doi: 10.1371/journal.pone.0045536 (PMC3448656; doi:10.1371/journal.pone.0045536)

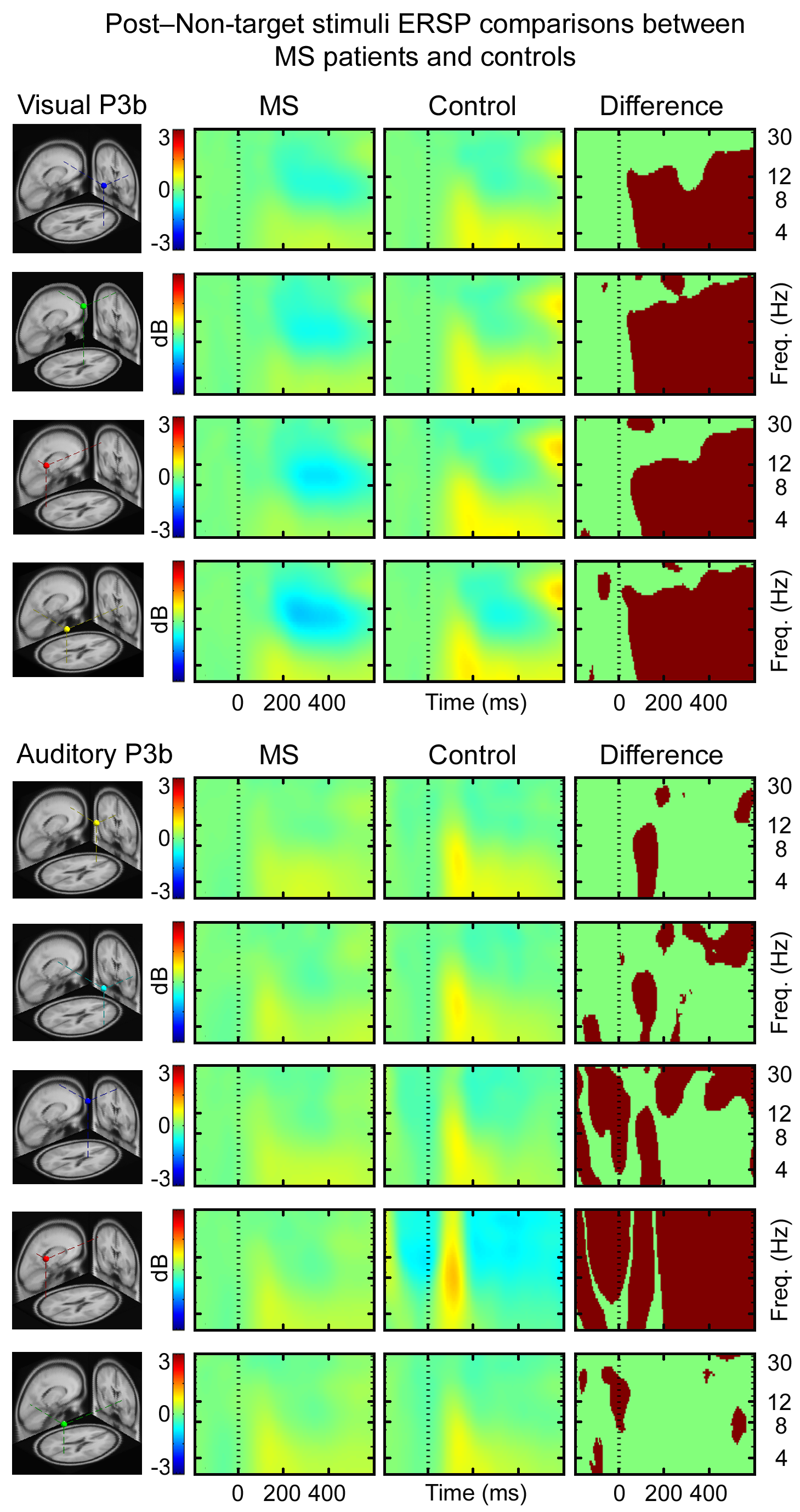

Supplement: Figure S1 — The mean ERSPs time-locked to the presentation of non-target stimuli in visual and auditory conditions are showing IC cluster mean differences in log spectral EEG power (dB) relative to log power in pre-stimulus EEG baseline. Red areas indicate an increase in power and blue areas a decrease in power. Statistical significance group and condition main effects, and interaction effect, are illustrated in red/green frames beside ERSP activation frames, in which red areas signify statistically significant (p<0.0125 for visual condition and p<0.01, controlled for multiple comparisons) differences between the MS patients and controls in time and in log spectral power. (TIF) [file pone.0045536.s001.tif]

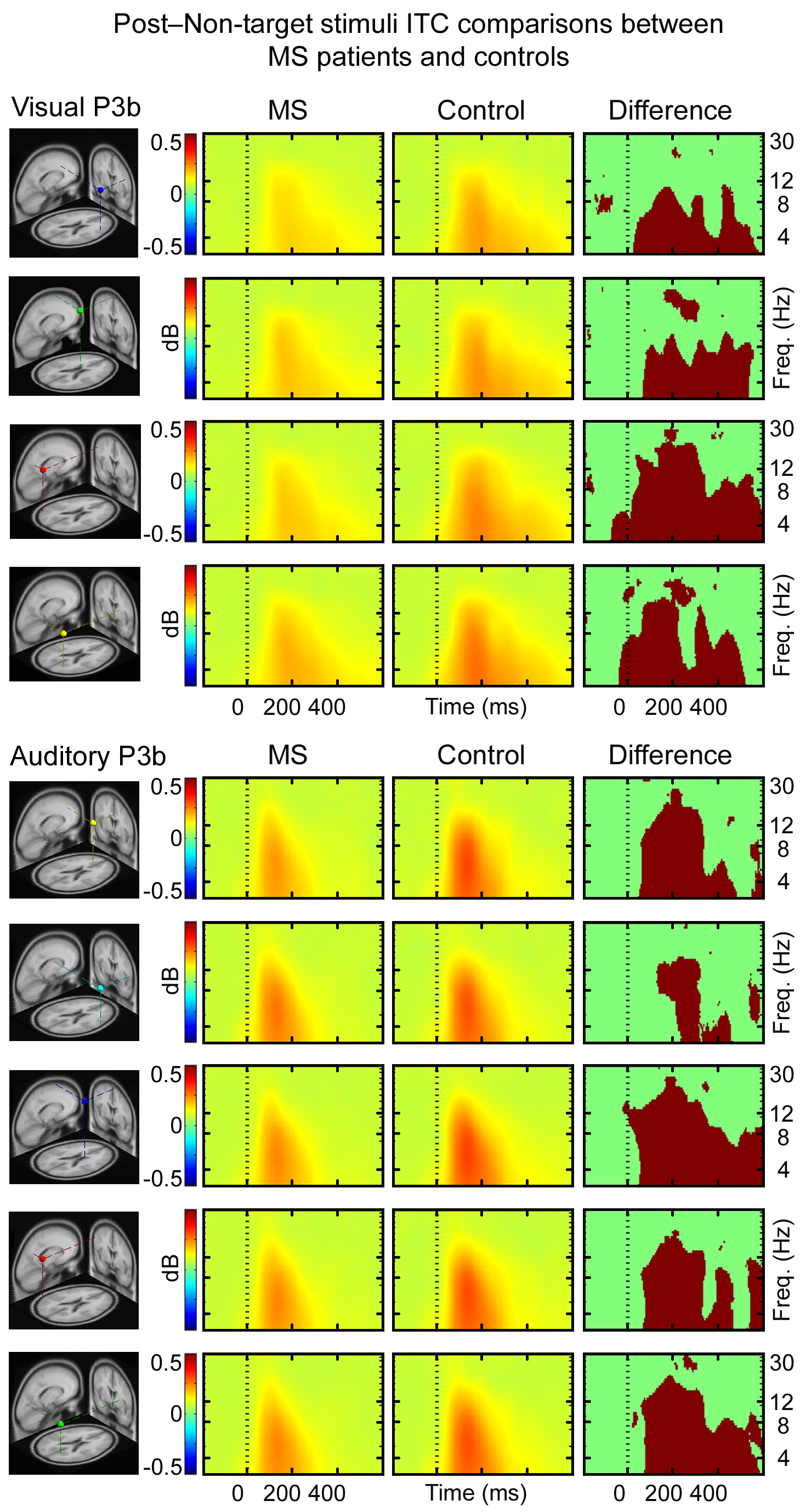

Supplement: Figure S2 — The ITC showing consistency between the trials and the degree of phase-locking to the non-target stimuli. Highest event-related phase consistencies for each condition and group are plotted in red, and lowest in green (range 0 to 1, no consistency to full consistency respectively). Statistical significance is illustrated by red/green frames, in which red areas signify statistically significant (p<0.0125 for visual condition and p<0.01 for auditory condition, controlled for multiple comparisons) differences between MS patients and controls in time and in log spectral power. (TIF) [file pone.0045536.s002.tif]

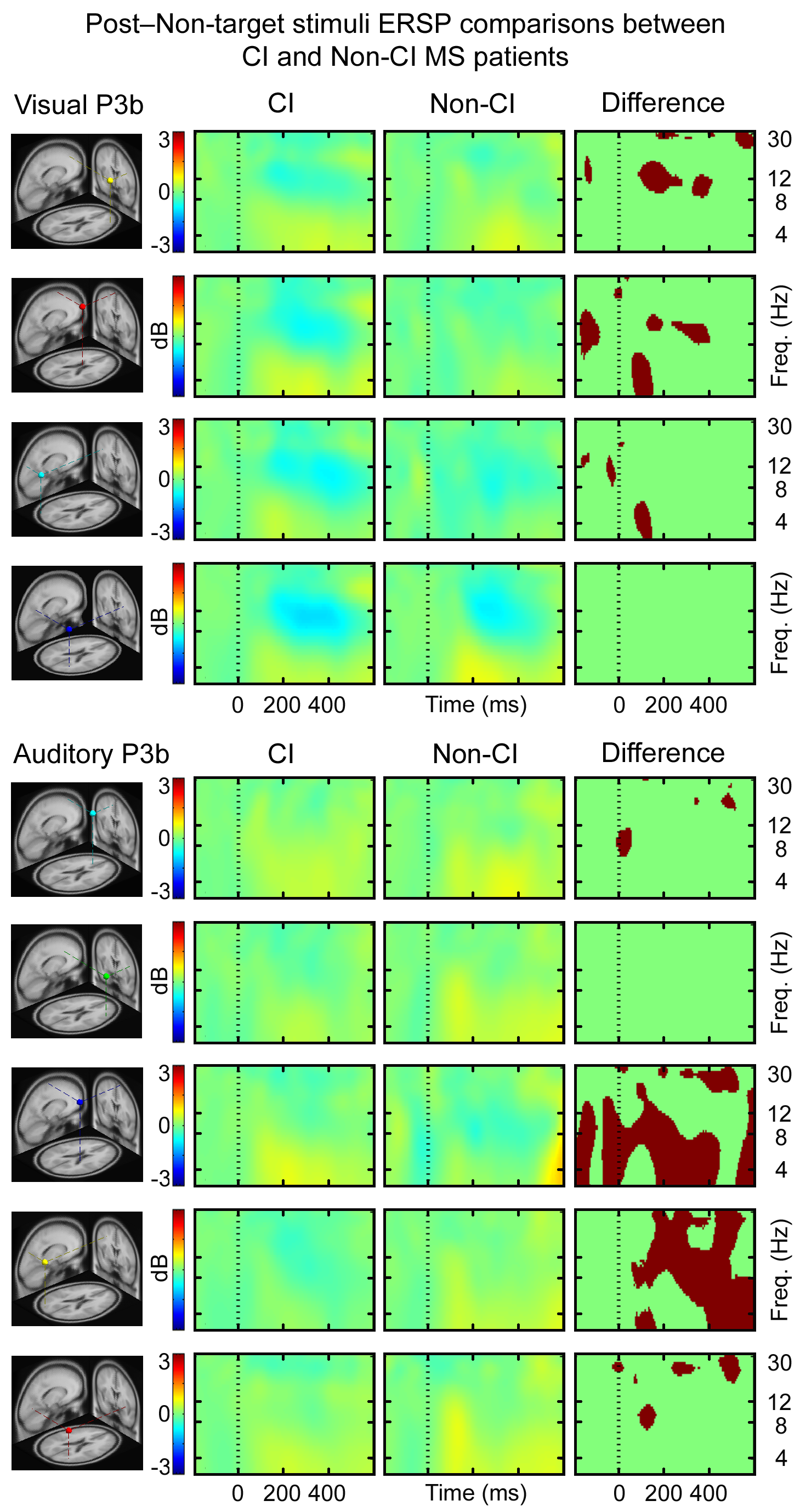

Supplement: Figure S3 — The mean ERSPs time-locked to the presentation of non-target stimuli in visual and auditory conditions are showing IC cluster mean differences in log spectral EEG power (dB) relative to log power in pre-stimulus EEG baseline. Red areas indicate an increase in power and blue areas a decrease in power. Statistical significance group and condition main effects, and interaction effect, are illustrated in red/green frames beside ERSP activation frames, in which red areas signify statistically significant (p<0.0125 for visual condition and p<0.01, controlled for multiple comparisons) differences between CI MS patients and the non-CI MS patients in time and in log spectral power. (TIF) [file pone.0045536.s003.tif]

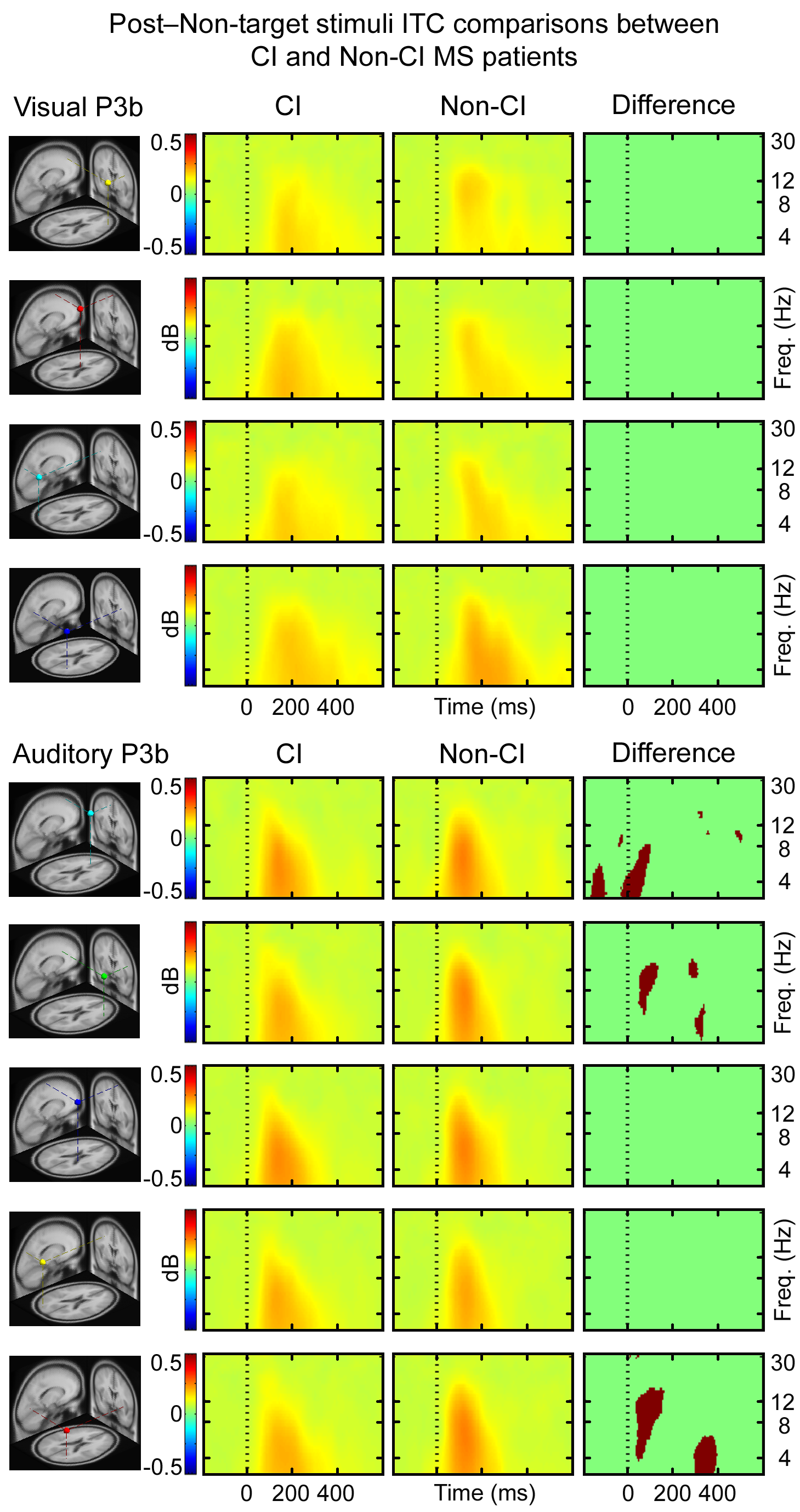

Supplement: Figure S4 — The ITC showing consistency between the trials and the degree of phase-locking to the non-target stimuli. Highest event-related phase consistencies for each condition and group are plotted in red, and lowest in green (range 0 to 1, no consistency to full consistency respectively). Statistical significance is illustrated by red/green frames, in which red areas signify statistically significant (p<0.0125 for visual condition and p<0.01 for auditory condition, controlled for multiple comparisons) differences between the CI MS patients and the non-CI MS patients in time and in log spectral power. (TIF) [file pone.0045536.s004.tif]

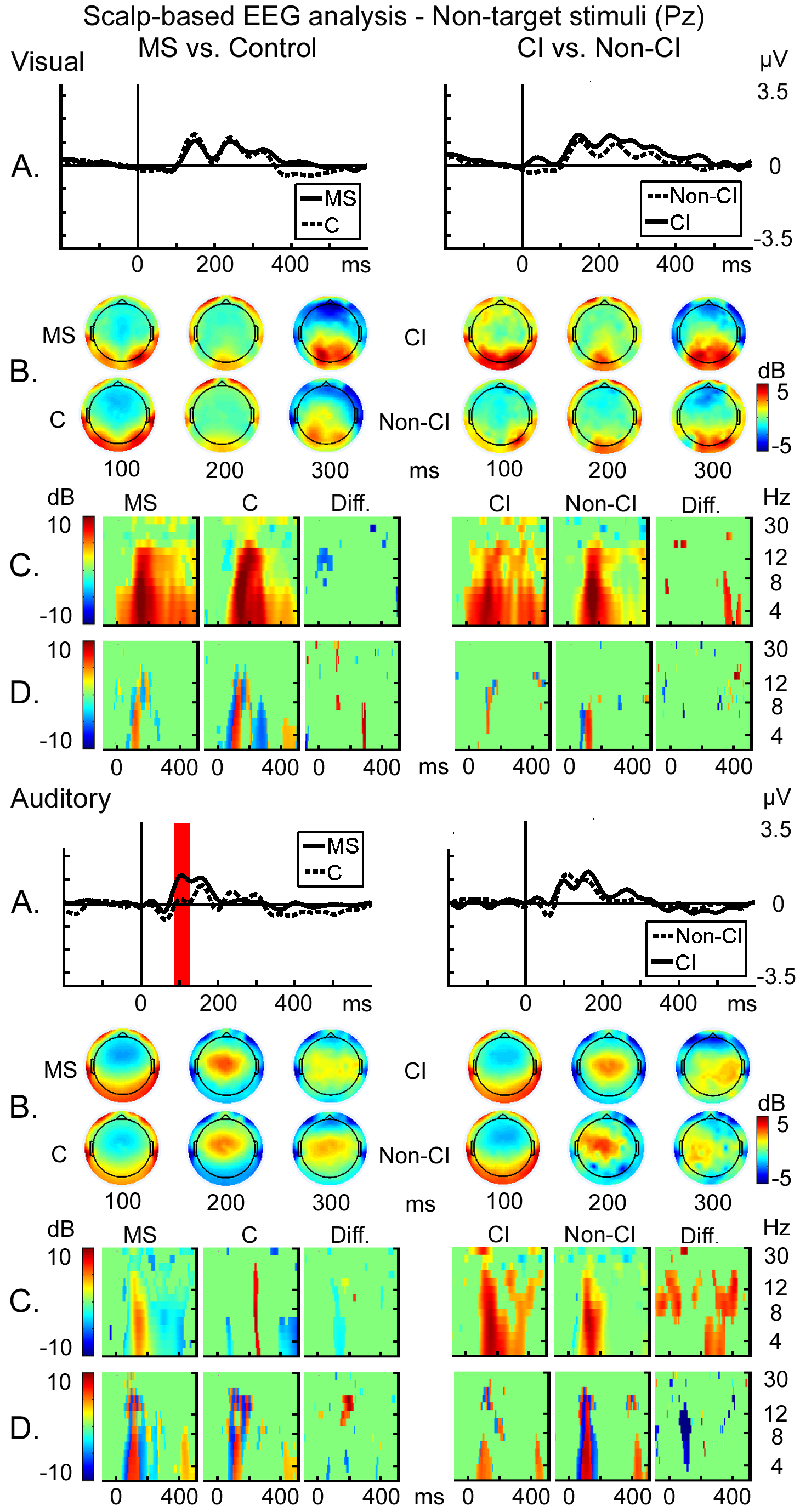

Supplement: Figure S5 — The scalp-based analysis of A) ERP, B) topography, C) ERSP, C) ITC of post–non-target activations at Pz in visual and auditory condition; comparison of MS patients and controls on the left side, and of CI and non-CI MS patients on the right side. Statistically significant (p<.05) differences in ERP indicated by red, and in the ERSP and ITC difference frames by non-green areas relative to time and log spectral power. Plots of the topographical responses 2-D circular view (looking down at the top of the head). Channel locations below head center are shown in a ‘skirt’ outside the cartoon head. Nose is at top of plot; left is left; right is right. (TIF) [file pone.0045536.s005.tif]

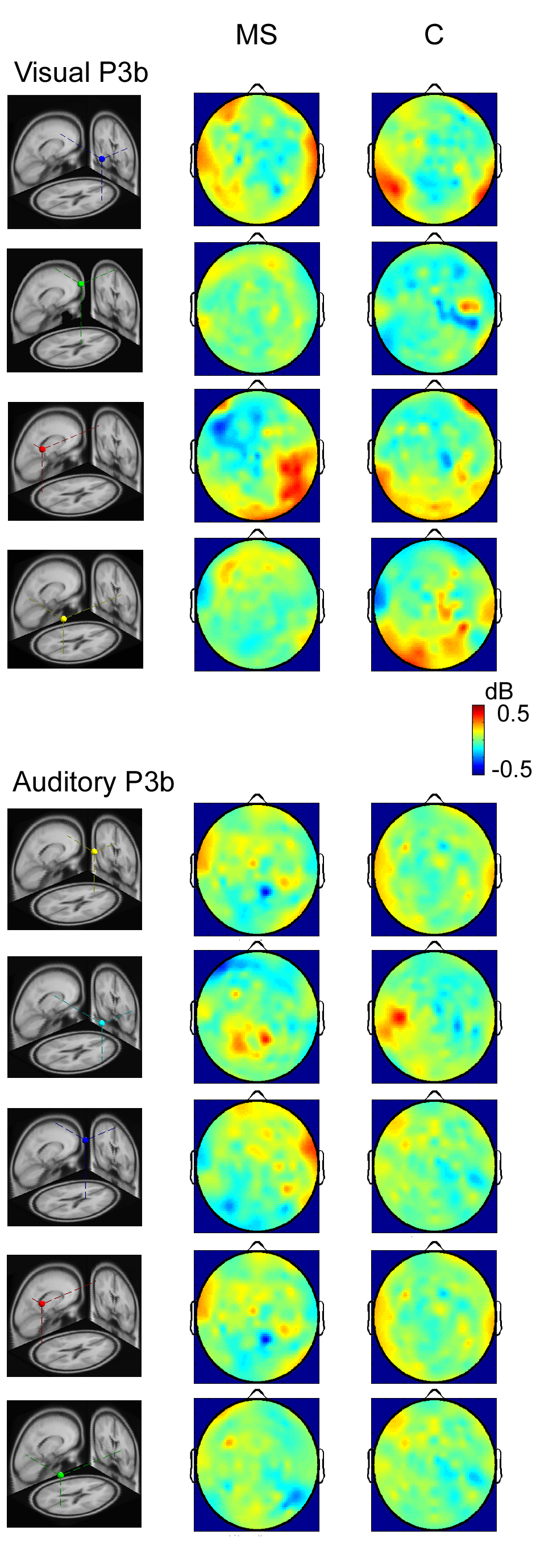

Supplement: Figure S6 — The grand mean topographies of MS patients and controls for each IC cluster post-target in visual and auditory conditions. (TIF) [file pone.0045536.s006.tif]

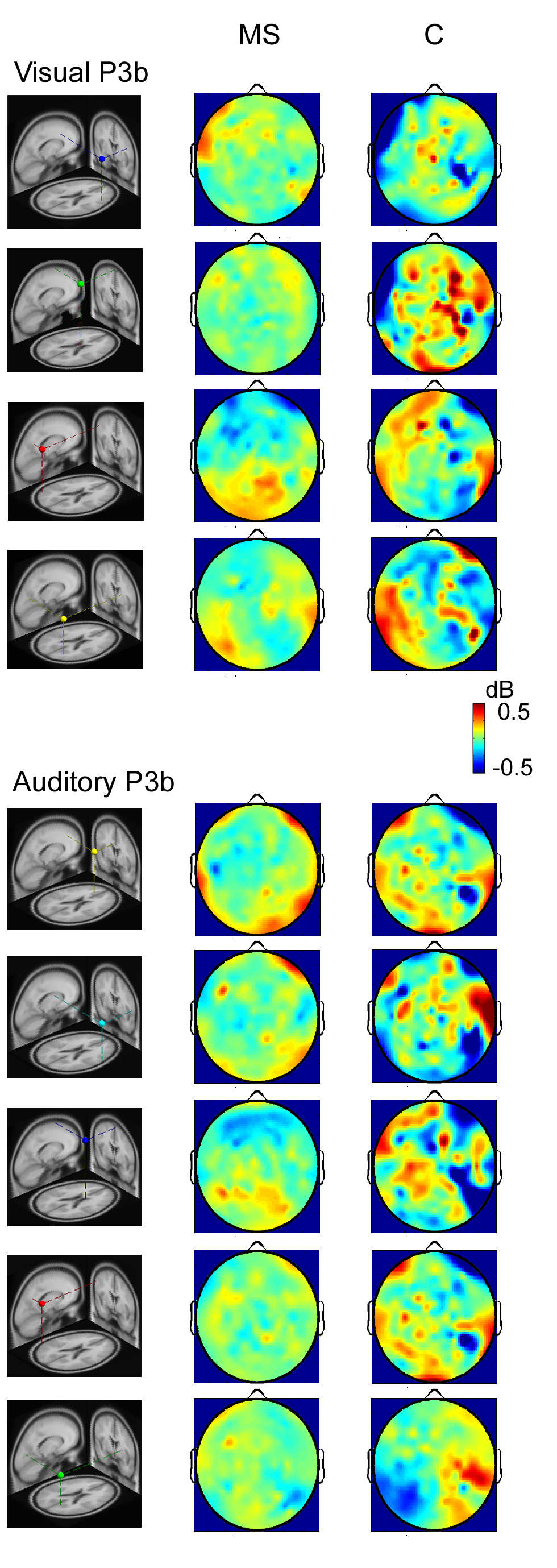

Supplement: Figure S7 — The grand mean topographies of MS patients and controls for each IC cluster post-non-target in visual and auditory conditions. (TIF) [file pone.0045536.s007.tif]

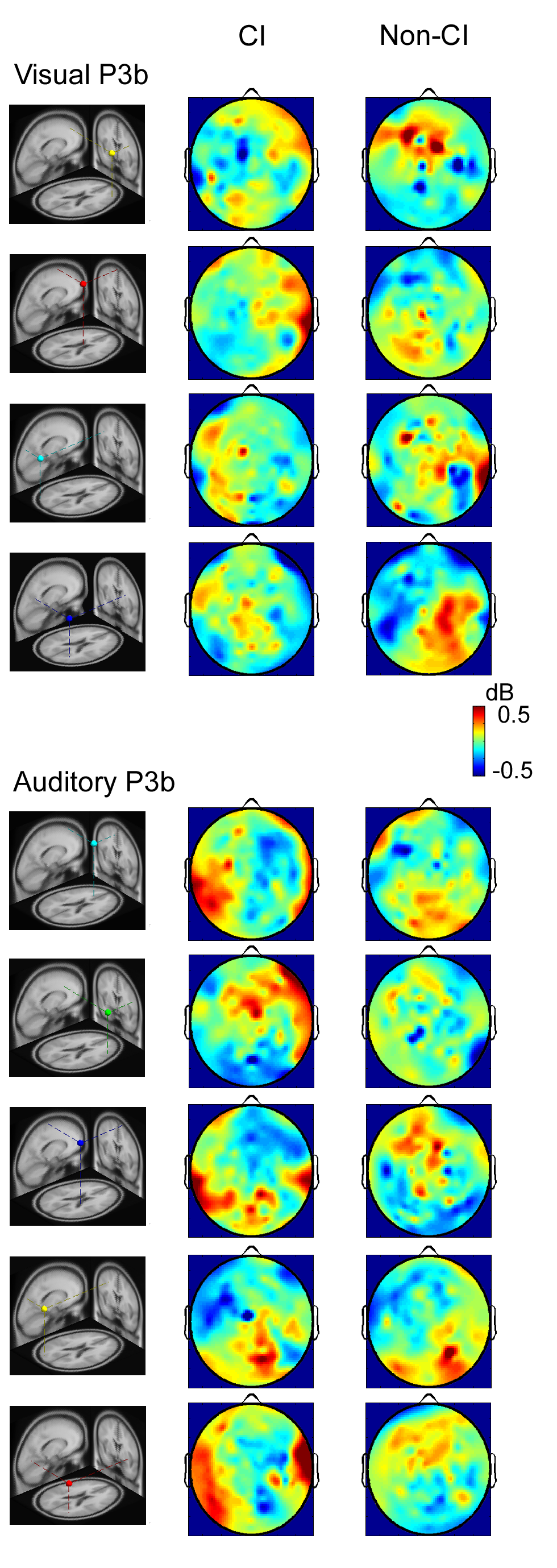

Supplement: Figure S8 — The grand mean topographies of CI and non-CI MS patients for each IC cluster post-target in visual and auditory conditions. (TIF) [file pone.0045536.s008.tif]

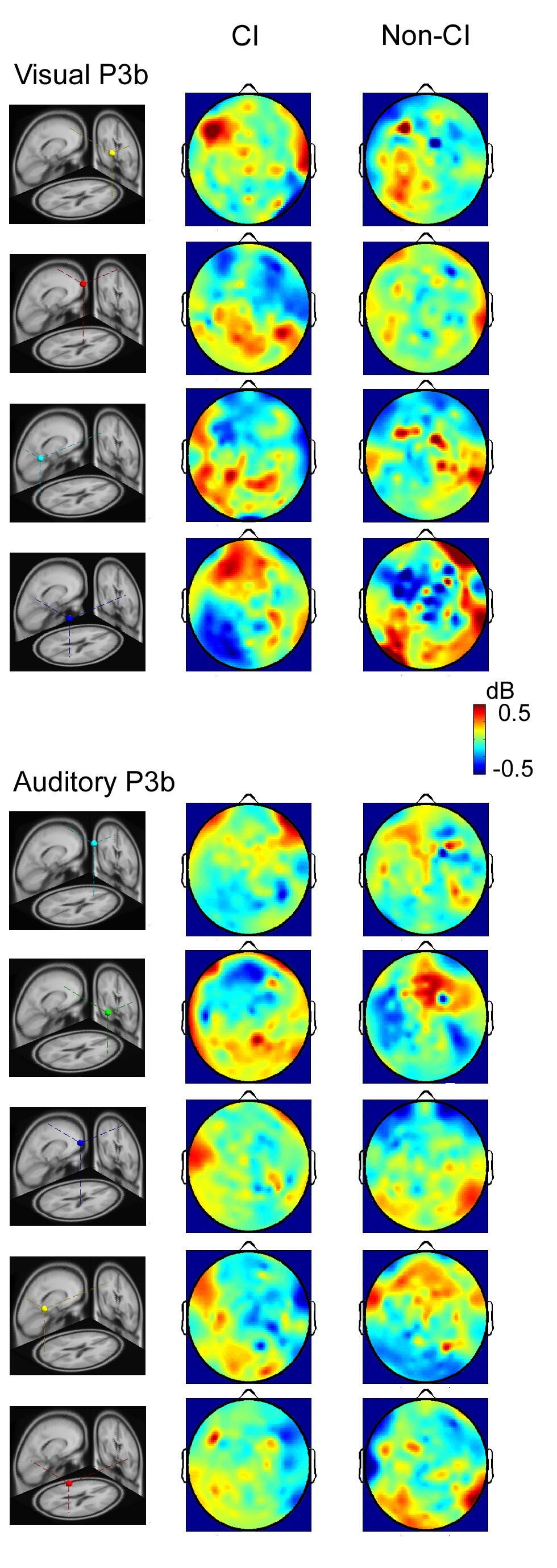

Supplement: Figure S9 — The grand mean topographies of CI and non-CI MS patients for each IC cluster post-non-target in visual and auditory conditions. (TIF) [file pone.0045536.s009.tif]

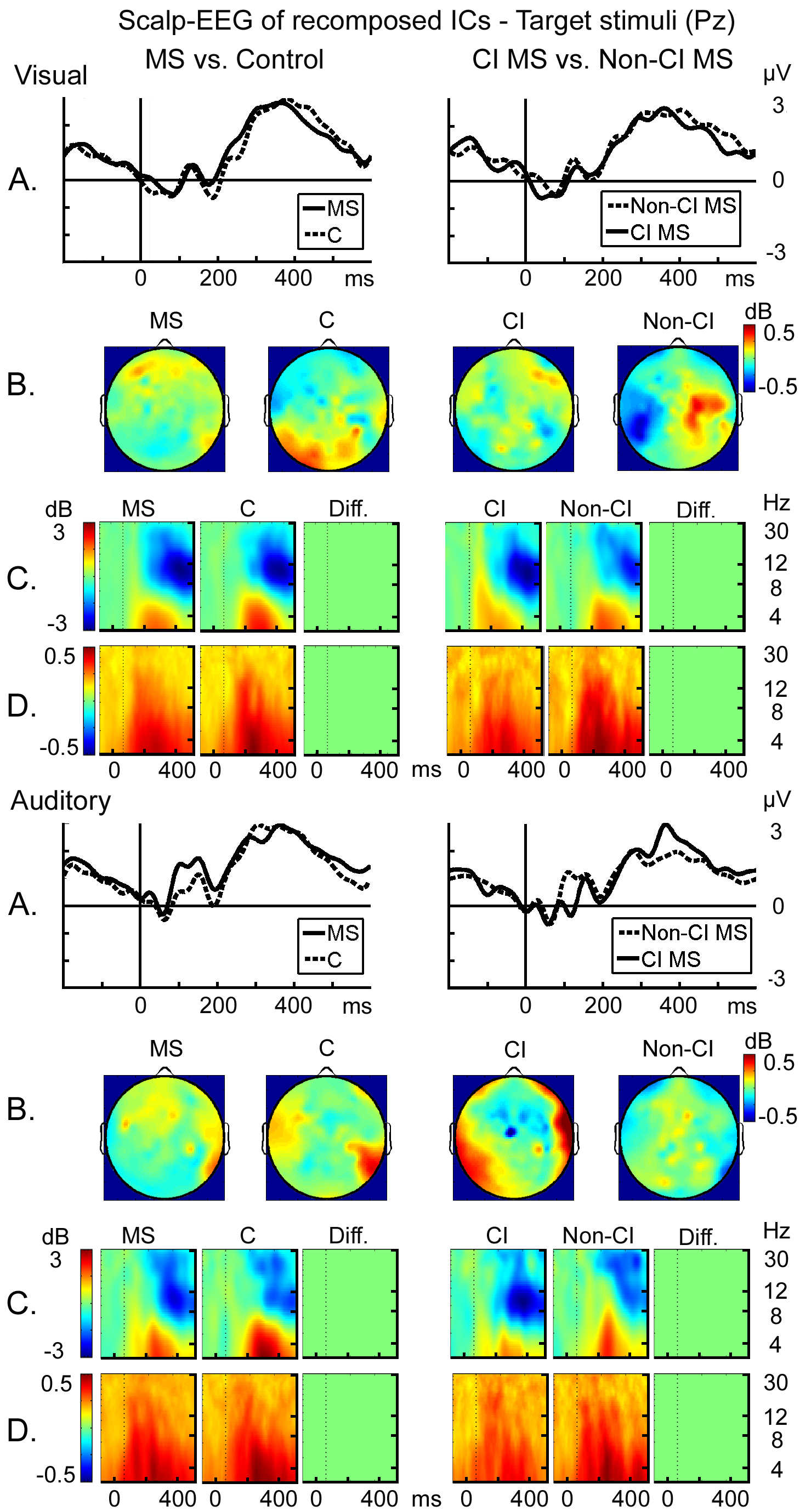

Supplement: Figure S10 — The EEG-scalp analysis of recomposed ICs included in the IC clustering analysis. A) ERP, B) mean topography, C) ERSP, D) ITC of post-target activations at Pz in visual and auditory condition; comparison of MS patients and controls on the left side, and of CI and non-CI MS patients on the right side. Statistically significant (p<.05, FDR corrected) differences in ERP indicated by red, and in the ERSP and ITC difference frames by non-green areas relative to time and log spectral power. (TIF) [file pone.0045536.s010.tif]

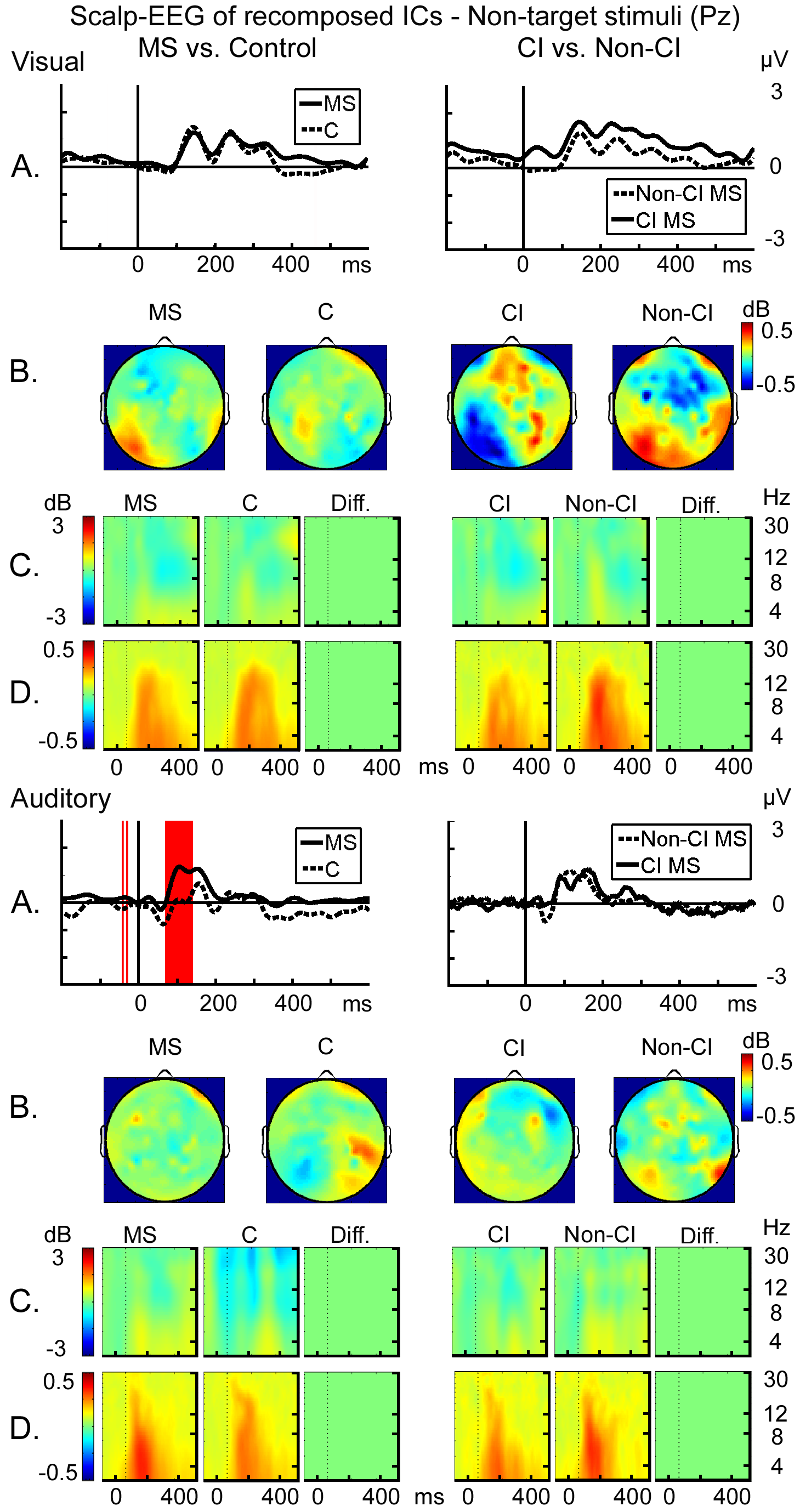

Supplement: Figure S11 — The EEG-scalp analysis of recomposed ICs included in the IC clustering analysis. A) ERP, B) mean topography, C) ERSP, D) ITC of post-non-target activations at Pz in visual and auditory condition; comparison of MS patients and controls on the left side, and of CI and non-CI MS patients on the right side. Statistically significant (p<.05, FDR corrected) differences in ERP indicated by red, and in the ERSP and ITC difference frames by non-green areas relative to time and log spectral power. (TIF) [file pone.0045536.s011.tif]
