# Supplementary material for: Only Low Frequency Event-Related EEG Activity Is Compromised in Multiple Sclerosis: Insights from an Independent Component Clustering Analysis
Source: PLoS One. 2012 Sep 21;7(9):e45536. doi: 10.1371/journal.pone.0045536 (PMC3448656; doi:10.1371/journal.pone.0045536)
Supplement: Table S1 — The number of subjects and independent components from each groups in each IC cluster. (DOCX) [file pone.0045536.s012.docx]

Table S1. The number of subjects and independent components from each groups in each IC cluster.

|  | Number of subjects | | | | Number of components | | | | | | |
| --- | --- | --- | --- | --- | --- | --- | --- | --- | --- | --- | --- |
|  |  | | | |  | | | | | | |
| **MS vs. C** | MS | C | | | MS | | | C | | |  |
| **Visual** |  | | | |  | | | | | | |
| Right frontal IC cluster | 38 | | 21 | | | | 305 | | 240 | |  |
| Central IC cluster | 35 | | 24 | | | | 389 | | 223 | |  |
| Right parietal IC cluster | 54 | | 38 | | | | 385 | | 362 | |  |
| Left parietal IC cluster | 35 | | 19 | | | | 268 | | 207 | |  |
| **Auditory** |  | | | |  | | | | | | |
| Frontal IC cluster | 43 | | 23 | | | | 322 | | 197 | |  |
| Right temporal IC cluster | 45 | | 25 | | | | 300 | | 218 | |  |
| Central IC cluster | 41 | | 24 | | | | 409 | | 274 | |  |
| Right parietal IC cluster | 54 | | 39 | | | | 397 | | 309 | |  |
| Left parietal IC cluster | 43 | | 22 | | | | 314 | | 260 | |  |
| **CI vs. Non-CI** | CI | Non-CI | | | CI | | | Non-CI | | |  |
| Right frontal IC cluster | 11 | | | 10 | | 62 | | | | 108 | |
| Central IC cluster | 11 | | | 11 | | 91 | | | | 136 | |
| Right parietal IC cluster | 18 | | | 17 | | 124 | | | | 91 | |
| Left parietal IC cluster | 11 | | | 9 | | 70 | | | | 69 | |
| **Auditory** |  | | | |  | | | | | | |
| Left frontal IC cluster | 14 | | | 13 | | 112 | | | | 99 | |
| Right frontal IC cluster | 15 | | | 13 | | 88 | | | | 131 | |
| Central IC cluster | 13 | | | 12 | | 108 | | | | 112 | |
| Right parietal IC cluster | 16 | | | 17 | | 112 | | | | 105 | |
| Left parietal IC cluster | 16 | | | 17 | | 139 | | | | 97 | |

Note. IC = independent component, MS = MS patients, C = controls, CI = CI MS patients, non-CI = non-CI MS patients
